# Galvano-Therapeutics

**Published:** 1874-03

**Authors:** 


Galvano-Therapeutics.—*A Revised reprint of a Report made to the Illinois State Medical Society in* 1873. Philadelphia: Lindsay & Blakiston, 1873. Octavo; Cloth, pp. 64. Price, $1.25.

The medical and surgical applications of Electricity and Galvanism are receiving renewed attention from the profession. It can scarcely be doubted that valuable results are yet to be developed from this potent agency for the relief and cure of human maladies. Those who imagine that there is little or no importance to be attached to the enlightened selection of the kind of current, its direction, and its quantity and intensity, commit a grave error, and may expect nothing but failure in their electrical ventures. These little manuals will be found very useful in making the proper discriminations.

